# Differential effects of attachment security on visual fixation to facial expressions of emotion in 14-month-old infants: an eye-tracking study

**DOI:** 10.3389/fpsyg.2024.1302657

**Published:** 2024-02-01

**Authors:** Joana L. Gonçalves, Marina Fuertes, Susana Silva, Pedro Lopes-dos-Santos, Fernando Ferreira-Santos

**Affiliations:** ^1^Center for Research in Psychology for Positive Development, Lusíada University, Porto, Portugal; ^2^Center for Psychology at University of Porto, Faculty of Psychology and Education Sciences, University of Porto, Porto, Portugal; ^3^Escola Superior de Educação, Instituto Politécnico de Lisboa, Lisboa, Portugal; ^4^Neurocognition and Language Research Group, Center for Psychology at University of Porto, Faculty of Psychology and Education Sciences, University of Porto, Porto, Portugal; ^5^Faculty of Psychology and Education Science, University of Porto, Porto, Portugal; ^6^Laboratory of Neuropsychophysiology, Faculty of Psychology and Education Science, University of Porto, Porto, Portugal

**Keywords:** attachment, information processing, eye-tracking, affect-biased attention, facial expression of emotion, infancy

## Abstract

**Introduction:**

Models of attachment and information processing suggest that the attention infants allocate to social information might occur in a schema-driven processing manner according to their attachment pattern. A major source of social information for infants consists of facial expressions of emotion. We tested for differences in attention to facial expressions and emotional discrimination between infants classified as securely attached (B), insecure-avoidant (A), and insecure-resistant (C).

**Methods:**

Sixty-one 14-month-old infants participated in the Strange Situation Procedure and an experimental task of Visual Habituation and Visual Paired-Comparison Task (VPC). In the Habituation phase, a Low-Arousal Happy face (habituation face) was presented followed by a VPC task of 6 trials composed of two contrasting emotional faces always involving the same actress: the one used in habituation (trial old face) and a new one (trial new face) portraying changes in *valence* (Low-Arousal Angry face), *arousal* (High-Arousal Happy face), or *valence + arousal* (High-Arousal Angry face). Measures of fixation time (FT) and number of fixations (FC) were obtained for the habituation face, the trial old face, the trial new face, and the difference between the trial old face and the trial new face using an eye-tracking system.

**Results:**

We found a higher FT and FC for the trial new face when compared with the trial old face, regardless of the emotional condition (*valence, arousal, valence + arousal* contrasts), suggesting that 14-month-old infants were able to discriminate different emotional faces. However, this effect differed according to attachment pattern: resistant-attached infants (C) had significantly higher FT and FC for the new face than patterns B and A, indicating they may remain hypervigilant toward emotional change. On the contrary, avoidant infants (A) revealed significantly longer looking times to the trial old face, suggesting overall avoidance of novel expressions and thus less sensitivity to emotional change.

**Discussion:**

Overall, these findings corroborate that attachment is associated with infants’ social information processing.

## 1 Introduction

Different authors, such as Cassidy (1994) and Bridges and Grolnick (1995), argue that infants learn to express and regulate their emotions, particularly negative ones, in a manner that allows their attachment needs to be met by their caregivers. For the most part, infants with a secure attachment experience parental sensitivity in response to a broad range of emotional signals, which promotes open and flexible communication of both positive and negative affect and flexible regulation of emotion based on the demands of the situation (de Wolff and van Ijzendoorn, 1997; Fuertes et al., 2006, 2009, 2020; Lucassen et al., 2011; Barbosa et al., 2019, 2021). Many insecure-avoidant infants may experience parental intrusiveness, which promotes the defensive minimization of affect, and fewer caregiver-oriented (e.g., proximity seeking, looking toward the caregiver, asking the caregiver for assistance) and more self-oriented (e.g., self-soothing, self-distraction) regulation behaviors to prevent additional controlling behavior (Isabella and Belsky, 1991; Swanson et al., 2000; Cantero and Cerezo, 2001; Fuertes et al., 2006, 2009, 2020; Barbosa et al., 2019, 2021). Insecure-resistant infants, in contrast, may experience maternal passivity or unresponsivity, which promotes the maximization or heightening of affect and frequent use of caregiver-oriented regulation behaviors to gain the caregiver’s attention (Cantero and Cerezo, 2001; Fuertes et al., 2006, 2009, 2020; Barbosa et al., 2019, 2021). These patterns, which serve different functions in the context of the parent-infant relationship, are thought to become internalized and then generalized to other contexts in which they may be less adaptive (Bowlby, 1969/1982; Ainsworth et al., 1978; Goldsmith and Alansky, 1987; Bretherton, 1990; Sroufe, 1996; de Wolff and van Ijzendoorn, 1997; Cassidy and Shaver, 1999; Crittenden, 2000; Bakermans-Kranenburg et al., 2003; Bretherton and Munholland, 2008; Beebe et al., 2010; Verhage et al., 2016).

Models of attachment and social information processing suggest that the attention allocated to salient social information, such as facial expressions, might occur in schema-driven processing, according to infants’ attachment patterns (Field and Lester, 2010; Dykas and Cassidy, 2011; Johnson and Chen, 2011; Morales et al., 2016; Gonçalves et al., 2023). Previous studies have shown that recurrent attachment-related experiences shape representational, physiological, and behavioral responses to emotional information (Pollak et al., 2000, 2001; Pollak and Kistler, 2002; Pollak and Sinha, 2002; Dykas and Cassidy, 2011; Vrtička et al., 2012; Vrtička and Vuilleumier, 2012; Cooke et al., 2016, 2018). To our knowledge, Peltola et al. (2015) were the first authors to investigate infants’ attention to facial expressions in association with attachment security, while others have addressed the relationship between caregiver-infant attachment security and infants’ visual preferences using other social–emotional stimuli (see Johnson et al., 2007, 2010; Biro et al., 2015, 2017). They used a longitudinal design and studied infants’ normative developmental bias to fearful faces at 7 months (Peltola et al., 2008, 2009a,b, 2011, 2013; Forssman et al., 2014a) as an antecedent of later attachment quality at 14 months (Peltola et al., 2015). Consistent with the models reviewed above, the main finding was a smaller attentional bias to fearful faces in infants with an insecure attachment pattern vs. secure infants, and this effect was most clearly associated with attachment disorganization, suggesting altered sensitivity to threat-related cues in infancy as a testable trait linking attachment disorganization to later behavioral outcomes (for a review about the outcomes of threat-related attention bias in socio-emotional development please see Pérez-Edgar et al., 2017; Burris et al., 2019a,b; Fu and Pérez-Edgar, 2019). In the same vein, but using neurophysiological measures, Peltola et al. (2018a,b), measured event-related potentials (ERPs) to investigate whether cortical responses to facial expressions of fear were associated with the development of secure and insecure patterns of infant-caregiver attachment during the first year. Based on previous findings showing reduced attentional biases to fearful faces in infants with insecure and disorganized attachment, the authors hypothesized that insecure and disorganized attachment would be associated with reduced ERP differentiation of fearful from non-fearful faces. ERPs to facial expressions were measured at 7 months of age and attachment was assessed at 14 months of age with the Strange Situation Procedure. The results revealed that occipitotemporal face-sensitive ERP responses particularly in the time range of the N290 component were related to attachment security at 14 months. Furthermore, only securely attached infants showed age-typical cortical discrimination of fearful from non-fearful faces at 7 months, whereas a similar pattern of ERP responses was not observed in infants with insecure and disorganized attachment. These results add to previous findings by suggesting that patterns of secure and insecure infant attachment are related to early-emerging differences in the perceptual processing of facial emotions, which could have implications for the development of social competence. More recently, Forslund et al. (2019) found that disorganized children (6 to 7-year-old children) showed lowered attention to facial expressions, a diminished ability to discriminate facial expressions, and elevated emotional reactivity.

However, the specific developmental pathways that link attachment and socio-emotional development are still a topic of research (Belsky, 1997, 2005; Belsky and Pluess, 2009; Ellis et al., 2011; Cassidy et al., 2013; Paschall and Mastergeorge, 2016; Groh et al., 2017; Slagt et al., 2018; Behrendt et al., 2019). In the present work, we expected to contribute to the line of research investigating the relation between the quality of early social–emotional experiences and social information processing in infancy (e.g., Striano et al., 2002; de Haan et al., 2004; Taylor-Colls and Pasco Fearon, 2015; Kungl et al., 2017; Morales et al., 2017; Kataja et al., 2018; Juvrud et al., 2019; Kataja et al., 2019), specifically how infants visually explore facial expressions of emotion. Our primary goal was to test the hypothesis of potential differences in attention to facial expressions and emotional discrimination (sensitivity to emotional change) between infants classified as securely attached (B), insecure-avoidant (A), and insecure-resistant (C), and between infants grouped as securely (B) or insecurely attached (non-B).

Following the literature reviewed, we identified four major limitations/literature gaps which were addressed in the present study. First, the existing developmental literature has focused on normative attentional patterns according to age, namely, age-expected attentional bias (e.g., to fearful faces at 7-months of age) (e.g., Peltola et al., 2015). In this study, we go beyond the developmental normative attention bias according to age, to consider general visual preferences and their emotional correlates. Second, previous studies have failed to study attachment in a discriminating way, only considering the secure vs. insecure attachment pattern contrast (e.g., Peltola et al., 2018a,b). Here, we consider not only secure vs. insecure but consider A-B-C attachment patterns. We compare all three primary attachment groups other than combining the resistant and avoidant infants into one insecure group, which is critically important as there are different theoretically-derived predictions about the pattern of attention to emotional stimuli that insecure-avoidant and insecure-resistant infants may utilize. Third, several studies have used behavioral measures such as looking time, typically measured by recording cumulative gaze to a whole visual scene, which has been one of the most used behavioral indices of infants’ cognitive and perceptual capabilities (e.g., Johnson, 2004). Here, we used an eye-tracking system that allows for much more accurate measurement of how attention is allocated than general measures of looking time, through greater spatial and temporal precision in quantifying eye movements than video encoding by human scorers (see Papageorgiou et al., 2014; Leppänen et al., 2015; Crawford et al., 2016) and including the analysis of two different measures of infants visual preferences—mean FT and mean FC—, following the suggestion of LoBue et al. (2020) of using multiple outcome measures in infant research. Fourth, most developmental studies of emotion perception are not designed to distinguish whether infants discriminate visual affective features, such as *valence* (pleasant vs. unpleasant) and *arousal* (high vs. low) (Barrett et al., 2019). They tend to mix arousal and *valence* contrasts, or to present *valence* contrasts ignoring their *arousal* levels, into impure comparisons that need to be disentangled. For instance, previous studies have compared a pleasant instance of emotion (smiling in happiness) with one that is intended to depict an unpleasant instance of emotion (e.g., scowling in anger, or gasping in fear), ignoring their levels of *arousal* (e.g., Montague and Walker-Andrews, 2001; Leppänen et al., 2007, 2009). In this sense, we will try to disentangle this by selecting facial stimuli that differ in emotional *valence* (happy, angry) and the level of *arousal* (low, high). Therefore, we aimed to analyze if the impact of attachment security on infants’ visual preferences was independent of the emotional contrast presented, by including in our experimental paradigm three different emotional contrasts, namely, *valence*, *arousal*, and *valence + arousal* (following recent debates in the field of Facial Expressions of Emotion; see Almeida et al., 2016; Barrett et al., 2019; Pereira et al., 2019).

Based on the literature reviewed above (Isabella and Belsky, 1991; Swanson et al., 2000; Cantero and Cerezo, 2001; Fuertes et al., 2006, 2009, 2020; Barbosa et al., 2019, 2021), we hypothesized that: (a) since insecure-avoidant infants may experience consistent maternal intrusiveness, they may learn to attend away from feelings of distress and/or social-emotional stimuli to inhibit or prevent expression of distress when frightened, developing an avoidance attentional biases to social stimuli—irrespective of emotional valence—as a function of early interactions with the mother, and (b) contrarily, insecure-resistant infants are likely to learn to attend excessively to social and/or emotional stimuli, developing a *hypervigilance* attentional bias to social stimuli, due to the higher prevalence of passive or unresponsive maternal behaviors, feeling a higher need to monitor the environment. However, except for the studies developed by Peltola et al. (2015, 2018a,b), no previous study has examined the association between the processing of facial expressions and attachment security in infants. Therefore, our specific aims were to evaluate (1) 14-month-old infants general emotion discrimination abilities (using eye-tracking visual parameters of Fixation Time and Fixation Count, FT and FC, hereafter); (2) their emotion discrimination (FT and FC) as a function of attachment pattern (A, B, C); and (3) their emotion discrimination (FT and FC) as a function of attachment security (secure vs. insecure, B vs. non-B).

## 2 Method

### 2.1 Participants

Sixty-five infants and their mothers were recruited from an ongoing longitudinal cohort study. Four infants were excluded due to unsuccessful eye-tracking calibration (*n* = 2), or due to fussiness, movement, or inattentiveness during the experiment (*n* = 2). The final sample included 61 infants (35 girls, mean age = 13.93 months, *SD* = 1.21, range [12.00–16.50], birth weight ≥ 2,400 g; mean birth weight = 3,035 g, *SD* = 458, range [2,400–3,945]) and their mothers (*M* = 31.91, *SD* = 3.54, range [23.00–37.00]). First-minute Apgar scores ranged from 7 to 9 (*M* = 8.86, *SD* = 0.41). All infants were born full-term (Gestational Age (GA) ≥ 37 weeks; mean GA = 38.55 weeks, *SD* = 1.18, range [37.00–41.30]), healthy, and clinically normal at delivery. Parental self-report and medical records indicated no signs of sensory or neurological abnormalities, nor other illnesses or congenital anomalies in the neonatal period. The infants were all from middle-class Caucasian families. At the time of testing, they were free of visual or neurological abnormalities. Mother-infant dyads were recruited after delivery at the *Unidade Local de Saúde de Matosinhos* (Local Health Unit of Matosinhos) and at the *Centro Hospitalar de São João* (Hospital Center of São João). Recruitment was authorized by both Hospital administration boards, based on favorable reports of their respective ethical committees. The present study was conducted according to the ethical guidelines presented in the Declaration of Helsinki. Written informed consent was obtained from both parents, before conducting any assessment or data collection.

All infants lived with both parents in the same household. Families were from urban, middle-class socioeconomic backgrounds. No parent had any known serious physical illness, psychiatric condition, or drug/alcohol addiction problems. Most mothers (*n* = 32, 53%) completed a College/University degree, while 24 (39%) completed the 12th year (upper secondary school level) and five (8%) attained the 9th year (third level of basic education). All mothers were Portuguese, married or living with their spouse, had a healthy gestation, and had no problems at delivery. For 34 mothers (56%), this was their first-born child. At the time of testing, all mothers were employed, healthy, and free of visual or neurological impairments.

### 2.2 Materials

**Figure 1:**
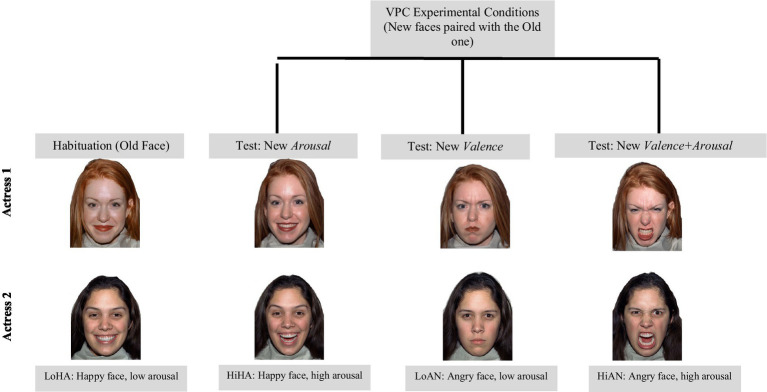
Stimulus materials used in the eye-tracking Visual Paired-Comparison (VPC) experiment. Due to image reproduction authorization restrictions, the images/actresses here presented are merely illustrative, although they are part of the NimStim Face Stimulus Set. Face stimuli here presented are merely illustrative, sourced from the NimStim set of Facial Expressions (Tottenham et al., 2009) and available to the scientific community at https://macbrain.org/resources/. All faces are from the Nim Stim data set publicly available for scientific research. The codes of the images for the two actresses used in our study from the NimStim set were: LoHA: 08_F_HA_O and 09_F_HA_O; HiHA: 08_F_HA_X and 09_F_HA_X; LoAN: 08_F_ AN_C and 09_F_AN_C; HiAN: 08_F_AN_O and 09_F_AN_O.

Stimulus materials for the eye-tracking experiment consisted of color images of female faces from two actresses exhibiting happy or angry expressions, taken from the NimStim Face Stimulus Set (Tottenham et al., 2009). Faces belonged to four conditions: High-Arousal Happy (HiHA), Low-Arousal Happy (LoHA), High-Arousal Angry (HiAN), and Low-Arousal Angry (LoAN). In our experiment, LoHA was used as the habituation face (and as the trial old face in the VPC experiment), with the other three representing changes in valence (LoAN), arousal (HiHA), or valence and arousal simultaneously (HiAN, see Figure 1). We only used the LoHa as the habituation face because it represents a valence (HA) and arousal (Lo) less likely to attract biased/reactive processing (baseline measure). This is because, in low-risk samples, infants’ early perceptual experiences with human faces are very likely dominated by low-arousal pleasant (happy) expressions from their caregivers, leading to perceptual expectations for these facial displays (for more detail, see Pereira et al., 2019). Stimulus selection was based on the following criteria: (1) the actress should be a European-American female; (2) happy and angry expressions with different levels of arousal should be available for the actor chosen; (3) finally, actresses should have at least four stimuli with recognition rate ≥ 0.80 (recognition rates in adults from Tottenham et al., 2009). Importantly, arousal ratings were examined to select an actress that had similar ratings for Low Happy (LoHA) and Low Angry (LoAN) stimuli, and also for High Happy (HiHA) and High Angry (HiAN) stimuli (arousal ratings in adults from Ferreira-Santos, 2013). Faces were presented with a uniform background (all white). Stimulus size and brightness were kept uniform using GIMP (Gimp, 2008).

In the test phase, the four faces were paired such that we had three types of contrasts between old face and new face for each actress: Arousal contrast: old face [LoHA] paired with new arousal face [HiHA], Valence contrast: old face [LoHA] paired with new valence face [LoAN], and Valence+Arousal contrast: old face [LoHA] paired with the new valence+arousal face [HiAN]. The “old face vs. new face” refers to the change of the facial expression or lack thereof, and not to the face’s identity; whereby the old/new contrast always involved the same actress. For each contrast (experimental condition), there were two versions: one with the new face on the left side, and the other with the new face on the right. Thus, each actress generated a set of six face pairs, each set defining a six-trial experimental protocol with three conditions. By switching the presentation order of 3 contrasts/conditions × 2 versions, we created 12 experimental protocols per actress (see Supplementary Table A1), and thus 12 × 2 = 24 protocols in total. Each infant was assigned to one of these (meaning that each infant only saw one actress throughout the experimental task).

### 2.3 Procedure

Potential participant mothers were contacted in the neonatal obstetric units within the first 72 h after the infants’ birth. The purpose and procedures of the study were explained, and mothers were invited to participate. If that was the case, informed consent was signed by the infants’ parents. Participant mothers were first administered a brief questionnaire to collect demographic information and data concerning infants’ perinatal health status was abstracted from medical records, to determine eligibility. When infants completed 14 months of age, a laboratory session was scheduled with the parents at the Faculty of Psychology of the University of Porto. The session began with the eye-tracking experiment, followed by the Strange Situation Procedure (SSP) (Ainsworth et al., 1978).

In the eye-tracking experiment, infants were accompanied by one of the parents, who was asked to help keep the infant’s attention focused on the stimuli during the session. Parents were told they could point to the computer screen or could say “*look at the face*,” but that they should not mention the emotion being displayed. Infants sat on a baby chair approximately 60 cm in front of a screen (47.5 × 29.5 cm), where they viewed the pictures of facial expressions. At this distance, each face corresponded to a horizontal visual angle of 9.52° and a vertical angle of 14.25° (both in habituation and in the VPC paradigm). Faces size was 10 cm × 15 cm and paired faces (in the VPC trials) were separated by a 12 cm interval, ear to ear. Infants’ mothers or fathers remained behind them with a hand on their shoulder, so that they would feel secure. Parents were asked to look above the screen to avoid any interference with infants’ gaze tracking. The infant’s body was stabilized with an Infant car seat with an arm support. Eye movements were recorded monocularly with a remote eye-tracking system (SMI RED 250^1^), at a 120 Hz sampling rate.

**Figure 2:**
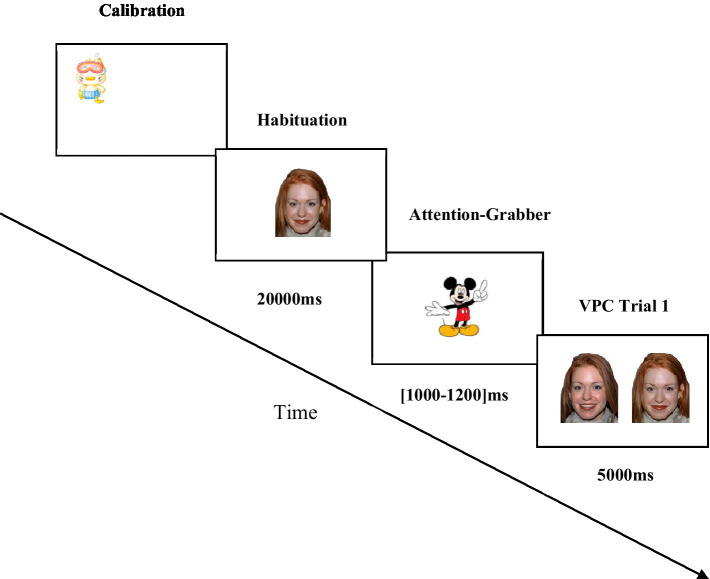
Experimental paradigm used in the eye-tracking session. Face stimuli here presented are merely illustrative, sourced from the NimStim set of Facial Expressions (Tottenham et al., 2009) and available to the scientific community at https://macbrain.org/resources/. All faces are from the Nim Stim data set publicly available for scientific research. The codes of the images for the two actresses used in our study from the NimStim set were: LoHA: 08_F_HA_O and 09_F_HA_O; HiHA: 08_F_HA_X and 09_F_HA_X; LoAN: 08_F_ AN_C and 09_F_AN_C; HiAN: 08_F_AN_O and 09_F_AN_O.

Stimuli were presented using the SMI Experiment Center (version 3.0). Each testing session began with a 5-point calibration procedure. The calibration point was an animation that appeared in each of the four corners of the computer monitor and the center of the screen, accompanied by a playful sound to capture the infant’s attention. Calibrations were considered successful when deviations were below 2°. Unsuccessful calibrations were reattempted twice. In case of repeated failure, the experiment would not run, and the participant was excluded from the experiment (two infants were excluded due to unsuccessful calibration as explained in the sample subsection). Each infant was assigned to one of the 24 available protocols (see Supplementary Table A1). Stimulus presentation was automated after a successful calibration. For all protocols (see Figure 2), each trial began with a Low Happy face (LoHA, habituation face) which was displayed in the center of the screen for 20s (see, e.g., Pascalis et al., 2002). The habituation phase was followed by the test phase with the six Visual Paired Comparison (VPC) trials. Each trial consisted of two emotional faces—the old face (similar to the habituation face), accompanied by a new face (HiHA, LoAN, or HiAN). Given the infants’ limited attention span, trials were organized into two blocks, three trials per block. Each trial was displayed for 5,000 ms (5 s). To maintain infants’ attention throughout the experiment and prevent fatigue, an attention-grabber was used. We used four different animated audiovisual stimuli accompanied by a piece of baby-Disney music (random duration between 1,000 ms and 1,200 ms).

After the eye-tracking experiment, mother-infant dyads participated in the Strange Situation Procedure (SSP) (Ainsworth et al., 1978). The SSP is a 21-min laboratory paradigm designed for assessing the infant’s attachment pattern. The paradigm involves a sequence of eight episodes designed to place mild but increasing levels of stress on the infant (i.e., being introduced to an unfamiliar playroom, interacting with an unfamiliar adult stranger, and brief separations from and reunions with the mother). By standard procedures, the SSP consisted of eight 3-min episodes, including two separations from and two reunions with the mother, and interaction with a female stranger.

### 2.4 Data analysis

#### 2.4.1 Preprocessing of eye-tracking data

The eye-tracking data quality in terms of attrition rate and proportion of valid trials had to be similar to previous infancy eye-tracking studies (i.e., attrition rate around 20–35% or lower, based on Watanabe et al., 2012; Ambrosini et al., 2013; Oakes and Ellis, 2013), and proportion of valid trials in each eye tracking task at greater than 70%, based on Forssman et al. (2014b) and Leppänen et al. (2015). Trials were inspected for artifacts, and those with more than 20% data loss were rejected (i.e., since trials were displayed for 5 s, only valid trials with signal processing for 4 s were retained). This resulted in the loss of two infants (as explained in the sample subsection). Only participants with all six valid trials (i.e., two valid trials for each of the three conditions) were included in the statistical analysis. Rectangular Areas Of Interest (AOIs) were created around each face - the habituation face as well as each face in VPC trials. For each AOI x Trial x Participant, BeGaze software (v. 3.0) provided measures of fixation time (ms) and fixation count (number of fixations). For VPC trials, we computed the difference between the old face and the new face, which was used as a dependent variable in the analysis.

#### 2.4.2 Classification of attachment patterns from SSP

Infants’ attachment behaviors were coded from video recordings, according to the attachment patterns (secure, insecure-avoidant, insecure-resistant) defined by Ainsworth et al. (1978). The scoring was made by two expert and reliable coders. For the four-way ABC classifications and presence vs. absence of a secure attachment (B vs. non-B), intercoder agreement was 92%. Different classifications were resolved in conference and consensus was achieved between the coders.

#### 2.4.3 Statistical analysis

Preliminary analyses were first carried out to evaluate the distributional properties of the study variables, including identifying potential outliers, kurtosis, and skewness, following the criteria recommended by Kline (1998), and to calculate descriptive statistics for the study variables. Before the aim-oriented analyses, we tested the normality of the variables with Kolmogorov–Smirnov statistics to decide on the tests to perform. For *t*-tests and ANOVAs, preliminary analyses were first carried out to check their assumptions. We ran Levene’s test for homogeneity of variances. Since the homogeneity of variance assumption was not violated, parametric statistics were used.

To test for successful emotion discrimination, we first conducted a one-sample *t*-test (*t* = 0) entering the value of the difference [trial old face-trial new face] for both mean fixation time and the mean fixation count, taking the average values of all three new faces. To test the main hypothesis, we ran a mixed ANOVA, with *Emotional Contrast* (Valence, Arousal, Valence+Arousal) as a within-subjects factor, and *Attachment Pattern* (A, B, C) as a between-subjects factor. An additional analysis considering only whether the attachment pattern was secure (B) or not (non-B) was also carried out. Finally, to ensure that infants’ patterns of emotion discrimination were due to attachment pattern *per se*, and not to potential differences in habituation time (learning) across attachment patterns, we compared the habituation time across the three attachment patterns. To that end, we ran a one-way ANOVA using the mean fixation time toward the habituation face as the dependent variable and infants’ attachment patterns as the independent variable.

All statistical analyses were carried out using SPSS, version 27. Findings were denoted as statistically significant using an alpha of ≤0.05.

## 3 Results

### 3.1 Attachment patterns

In the final sample of 61 infants, 40 infants (66%) were classified as securely attached (B), while 21 were classified as insecurely attached, of which 11 (18%) were classified as insecure-avoidant (A), and 10 (16%) insecure-resistant (C). No infants met the criteria to be classified as disorganized (i.e., had scores higher than 5 in disorganization behaviors) (Main and Solomon, 1990). Cohen’s kappa coefficient for ABC classification (0.90) indicated excellent intercoder reliability. The final scores for discrepant cases were discussed and agreed upon by conferencing with an expert Strange Situation coder.

#### 3.1.1 General emotion discrimination abilities

We found significantly higher mean fixation time and fixation count for the trial new face when compared with the trial old face (Table 1) regardless of the emotional condition (*valence, arousal, valence + arousal* contrasts): *t*(60) = −2.30, *p* < 0.05, *d* = 0.35 and *t*(60) = −2.03, *p* < 0.05, *d* = 0.31, for FT and FC, respectively. This suggests that 14-month-old infants were able to discriminate emotional faces based on novelty preference.

**Table 1:** Infants’ descriptive statistics for eye-tracking data: habituation, overall difference [trial old face-trial new face], and difference [trial old face-trial new face] according to valence, arousal, and valence+arousal.

	Mean FT (ms) (SD)	Mean FC (SD)
**Habituation**		
Habituation Face (LoHA)	8012.44 (3925.01)	19.49 (9.61)
**VPC**		
Trial Old Face (LoHA)	1515.99 (605.99)	5.21 (2.50)
Trial New Face (LoAN)	1751.95 (765.17)	5.95 (2.57)
Trial New Face (HiHA)	1967.80 (989.62)	6.42 (3.04)
Trial New Face (HiAN)	1865.13 (726.02)	5.55 (2.22)
Trial New Face (average LoAN, HiHA, HiAN)	1745.72 (660.32)	5.97 (2.33)
Trial Difference [Trial Old Face-Trial New Face]	−232.05 (662.52)	−0.77 (2.48)
**Emotional condition**		
Valence	−179.52 (984.38)	−0.66 (3.42)
Arousal	−473.39(1238.64)	−1.74 (3.27)
Valence + Arousal	−300.00 (950.21)	−0.94 (3.96)

VPC, Visual Paired-Comparison; FT, Fixation Time; FC, Fixation Count; LoHA, Low-Arousal Happy Valence face; LoAN, Low-Arousal Angry Valence face; HiHA, High-Arousal Happy Valence Face; HiAN, High-Arousal Angry Valence face.

#### 3.1.2 Emotion discrimination as a function of attachment pattern (A, B, C)

**Figure 3:**
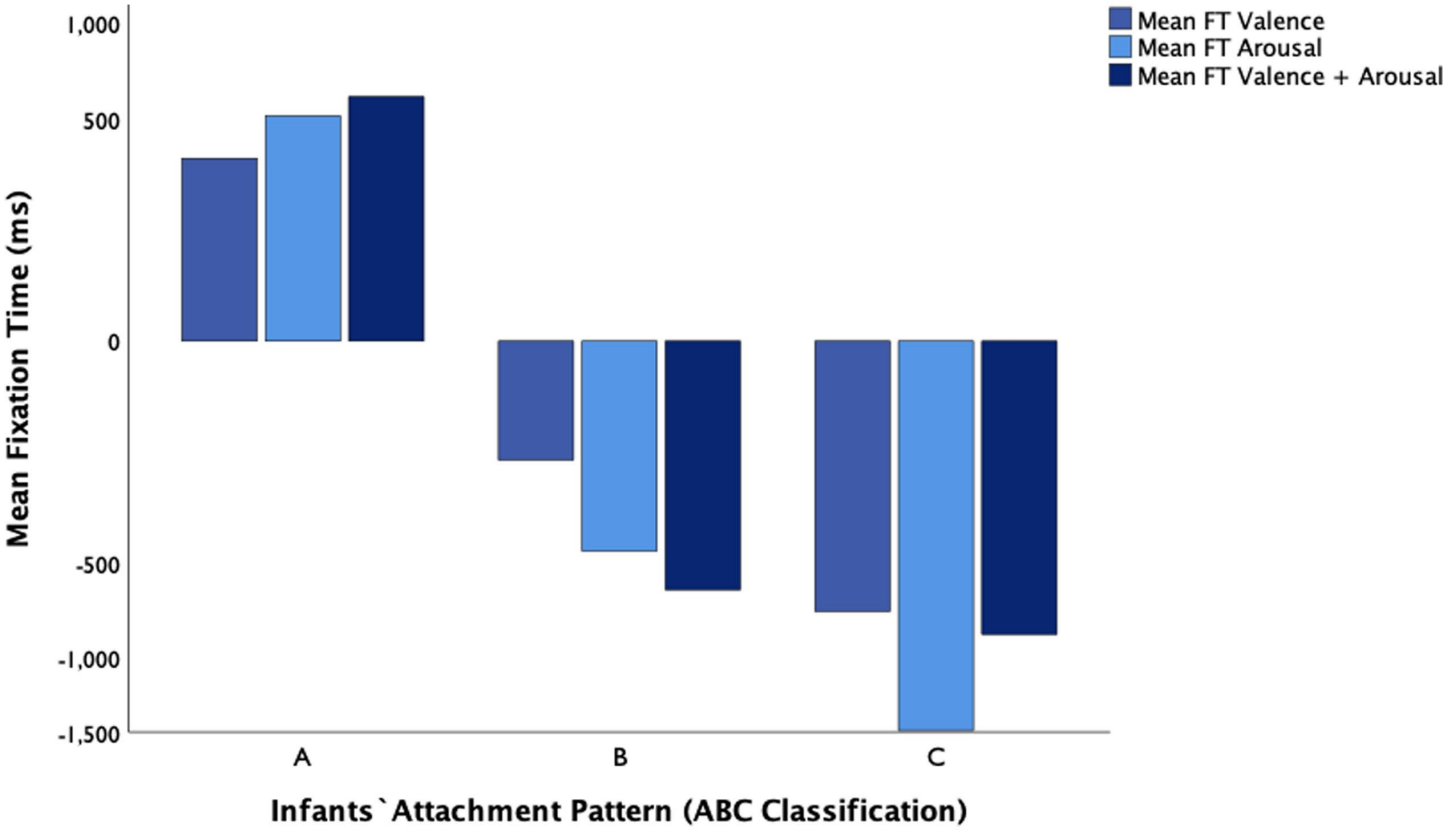
Mean FT (ms) for valence, arousal, and valence+arousal according to infants’ attachment pattern classification (A, B, C).

**Figure 4:**
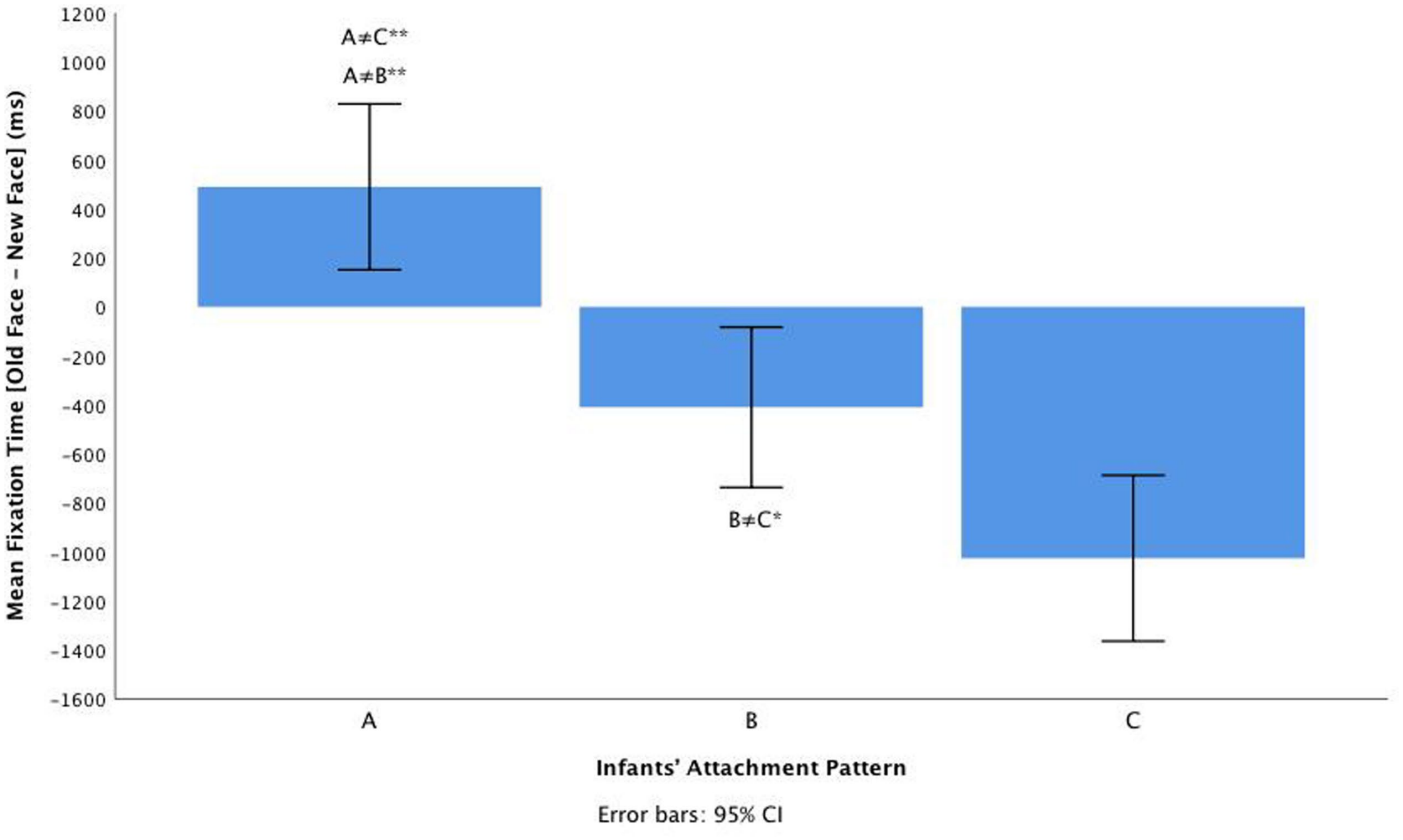
The main effect of attachment classification (A, B, C) on infants mean Fixation Time [trial old face – trial new face], *p < 0.05; **p < 0.001.

The mixed-factors ANOVA with mean fixation time as the dependent variable showed that the main effect of the emotional condition (trial old face—trial new face per valence, arousal, and valence+arousal, Table 1) was not significant: *F*(2, 116) = 1.79, *p* = 0.174, η^2^*_p_* = 0.043. Infants showed sensitivity to the new face, independently of the novel emotional condition (*valence, arousal, or valence + arousal* contrasts). There was, however, a significant large main effect of attachment pattern on the mean fixation time: *F*(2, 58) = 20.72, *p* < 0.001, η^2^*_p_* = 0.509 (see Figure 3 for descriptives). Tukey HSD *post hoc* tests revealed that there were statistically significant differences between insecure-avoidant infants (*M =* 489.63, *SD =* 167.51) and insecure-resistant infants (*M =* −1026.06, *SD =* 167.51, *p* < 0.001); between secure infants (*M =* −409.89, *SD =* 161.83) and insecure-resistant (*p* = 0.012); and between infants insecure-avoidant and secure infants (*p* < 0.001). Overall, mean FT differences [trial old face-trial new face] were larger for the insecure-resistant pattern (C) compared to both insecure-avoidant (A) and secure (B) patterns, indicating that children classified with an insecure-resistant attachment pattern remain hypervigilant toward emotional change (see Figure 4). The interaction between emotional contrast and attachment pattern for mean FT was not significant: *F*(4, 116) = 2.31, *p* = 0.065, η^2^*_p_* = 0.103.

**Figure 5:**
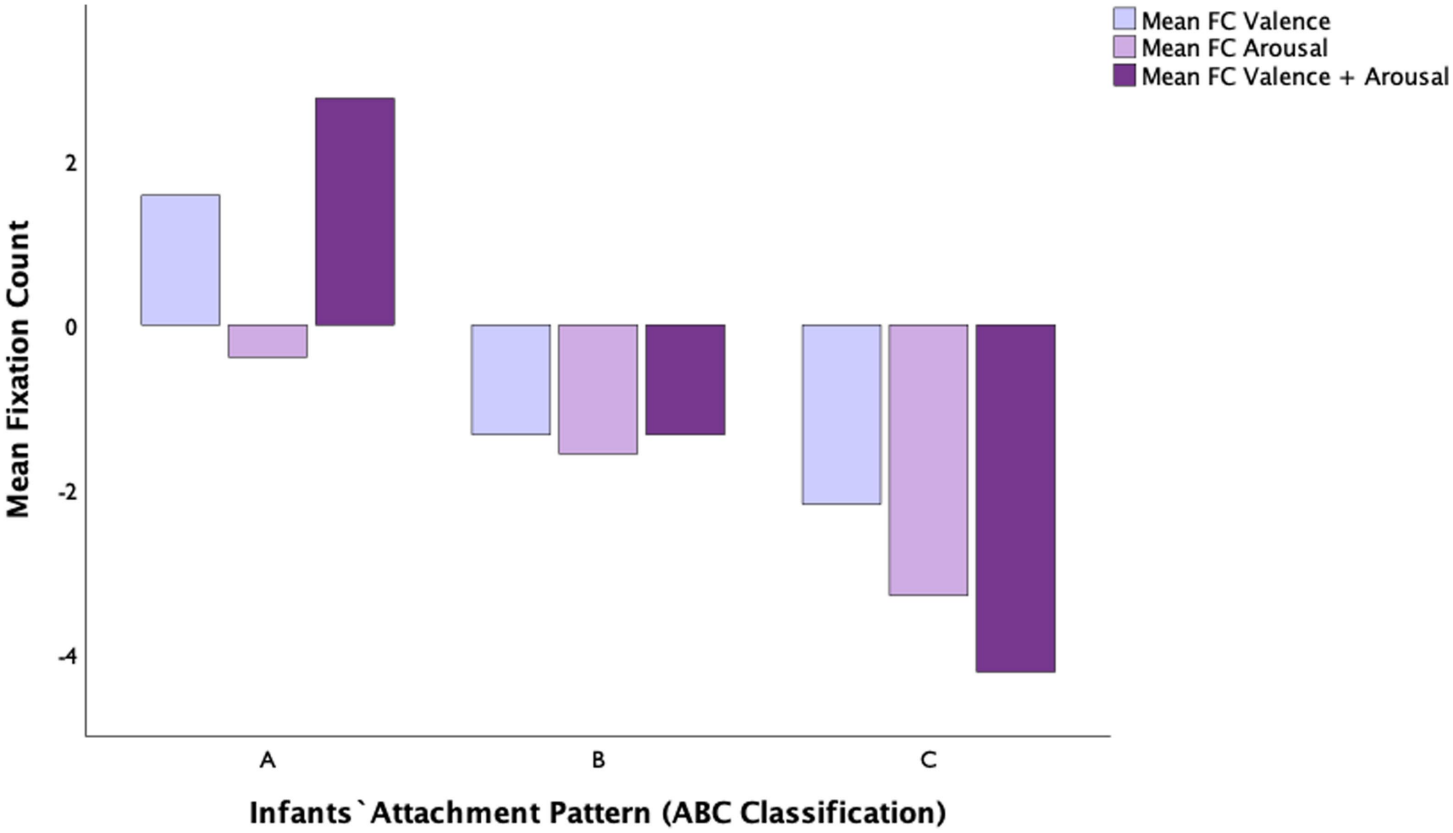
Mean FC for valence, arousal, and valence+arousal according to infants’ attachment pattern classification (A, B, C).

**Figure 6:**
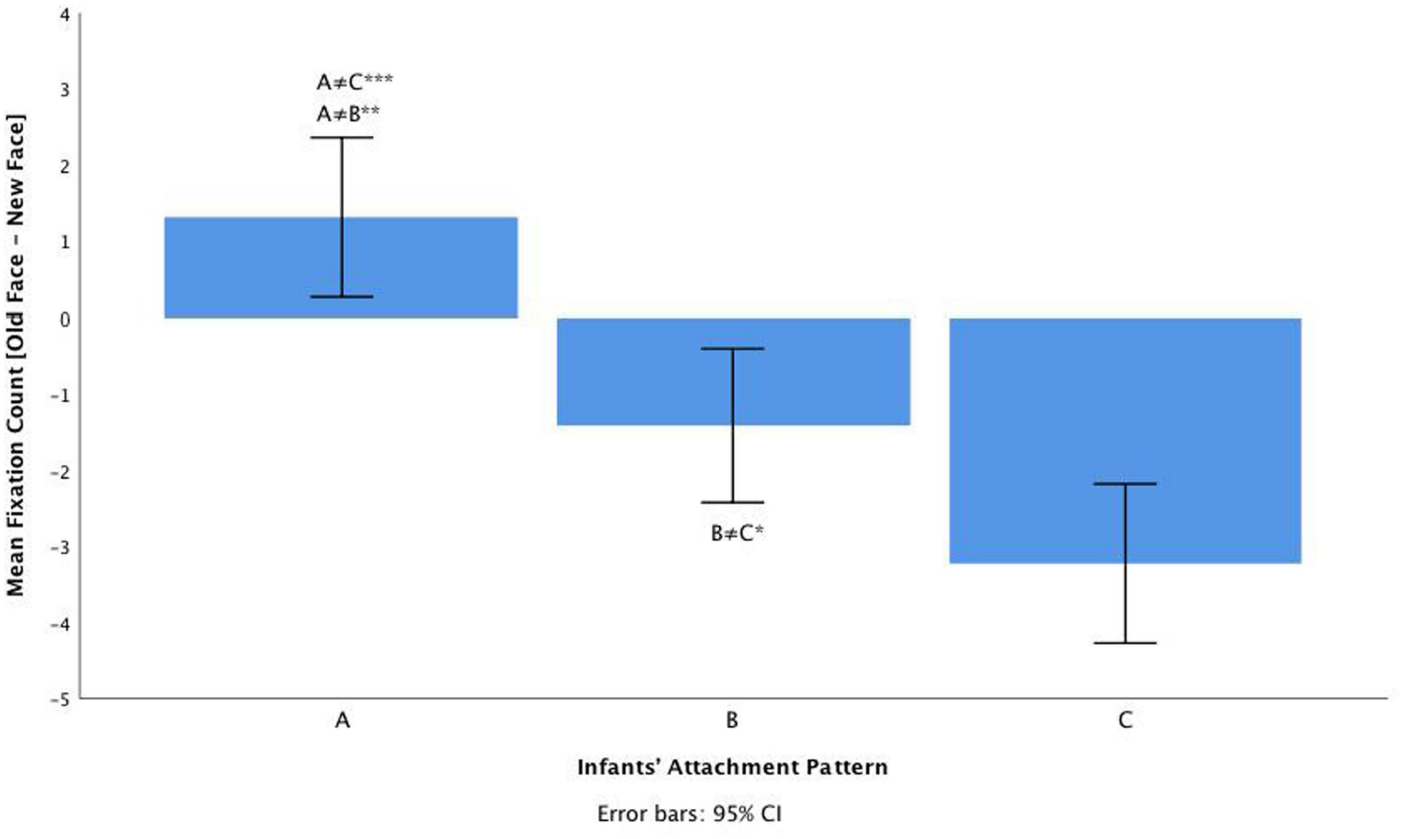
The main effect of attachment classification (A, B, C) on infants mean Fixation Count [trial old face – trial new face], *p < 0.05; **p < 0.01; ***p < 0.001.

Concerning fixation count as a dependent variable (Table 1), we found no significant main effect of emotional contrast [*F*(2, 116) = 1.77, *p* = 0.18, η^2^*_p_* = 0.042]. There was, however, a significant large main effect of attachment pattern on the mean fixation count: *F*(2, 58) = 19.43, *p* < 0.001, η^2^*_p_* = 0.493 (see Figure 5 for descriptives). Tukey HSD *post hoc* tests revealed that there were statistically significant differences between infants A (*M =* 1.31, *SD =* 0.52) and C (*M =* −3.23, *SD =* 0.52, *p* < 0.001); between infants B (*M =* −1.41, *SD =* 0.50) and C (*p* < 0.05); and between infants A and B (*p* < 0.01). Overall, mean FC differences [trial old face-trial new face] were larger for pattern C compared to both A and B, indicating that C children remain hypervigilant toward emotional change (see Figure 6). The interaction between emotional contrast and attachment pattern for mean FC was not significant: [*F*(4, 116) = 2.30, *p* = 0.066, η^2^*_p_* = 0.103].

#### 3.1.3 Emotion discrimination as a function of attachment security (B vs. non-B)

Using two levels for attachment pattern (secure vs. insecure, B vs. non-B), we found no significant main effects of emotional contrast [*F*(2, 118) = 1.61, *p* = 0.207, η^2^*_p_* = 0.038], attachment pattern [*F*(1, 59) = 0.253, *p* = 0.618, η^2^*_p_* = 0.006], nor any interaction effect between the two [*F*(2, 118) = 1.68, *p* = 0.192, η^2^*_p_* = 0.039] on mean fixation time.

The same tendency of no effect was found for fixation count as the dependent variable [emotional contrast: *F*(2, 118) = 0.975, *p* = 0.382, η^2^*_p_* = 0.023, attachment pattern: *F*(1, 59) = 0.279, *p* = 0.600, η^2^*_p_* = 0.006; interaction: *F*(2, 118) = 0.504, *p* = 0.606, η^2^*_p_* = 0.012].

#### 3.1.4 Fixation time in habituation across attachment patterns (control analysis)

A One-Way ANOVA revealed that the effect of attachment pattern on the mean Fixation Time for the habituation face was not significant at the *p* < 0.05 level [*F*(2, 60) = 1.25, *p* = 0.514, η^2^*_p_* = 0.012]. Therefore, the increased sensitivity of infants classified with an insecure-resistant attachment pattern to new faces does not seem to result from increased habituation time.

## 4 Discussion

In the present study, we found significantly higher mean FT and FC for the new face when compared with the old face, evaluated in the 6 experimental VPC trials, regardless of emotional condition (*valence, arousal, valence + arousal* contrasts), indicating that 14-month-old infants can discriminate novel emotional expressions. This finding was expected according to the developmental literature regarding the ontogeny of facial emotion processing, where behavioral studies have shown that infants’ ability to visually discriminate emotional expressions emerges around 5–7 months of age (e.g., Nelson and Dolgin, 1985; Nelson, 1987; de Haan and Nelson, 1998; de Haan, 2001; Kotsoni et al., 2001; Frank et al., 2009; Leppänen and Nelson, 2009; Frank et al., 2014).

Models of attachment and socio-emotional information processing have indicated that infants’ security in the primary caregiver availability and responsivity might influence the attention allocated to socio-emotional stimuli, namely, facial expressions of emotion (Field and Lester, 2010; Dykas and Cassidy, 2011; Johnson and Chen, 2011; Morales et al., 2016). Indeed, we found a significant main effect of attachment pattern on infants’ mean fixation time toward facial expressions emotional change, independent of the nature of that facial expression change (in each emotional contrast), suggesting that attachment is related to social information processing. The present results converge to suggest that patterns of secure and insecure infant attachment are related to differences in perceiving and attending to facial emotions, as has also been suggested in studies with children (e.g., Laible and Thompson, 1998; Pollak and Kistler, 2002; Pollak and Sinha, 2002; Bosmans et al., 2007; Steele et al., 2008; Vandevivere et al., 2014; Peltola et al., 2015; Forslund et al., 2017; Meinz et al., 2017; Peltola et al., 2018a,b), adolescents (e.g., Cassidy et al., 2003), and adults (e.g., Mikulincer, 1998; Mikulincer et al., 2000, 2002; Dewitte et al., 2007; Dewitte and De Houwer, 2008; Edelstein and Gillath, 2008).

Furthermore, *post hoc* tests revealed that there were significant differences between infants insecure-avoidant (A) and insecure-resistant (C), and between secure infants (B) and insecure-resistant (C). Overall, differences [old face-new face] were larger for attachment pattern C compared to both A and B, indicating that insecure-resistant children remain hypervigilant toward emotional change. Insecure-resistant infants show a preference for the new face, through a more negative value for the mean difference in the contrast [old face-new face] for both FT and FC, revealing a higher sensitivity to the novelty effect (an increased sensitivity to faces and facial expressions of emotion). Insecure-resistant infants’ attention deployment can be characterized by an *overall hypervigilance* visual tendency, as they seem to be more aware or hypervigilant to the new stimulus, suggesting a higher facial emotional discrimination (sensitivity to emotional change). The group of resistant infants is the only group that, compared with their counterparts, always prefers to look at the new face compared with the old face (across the three emotional contrasts), showing heightened attention toward the new stimuli.

Contrary to the hypervigilant pattern of emotion processing that characterized the resistant attachment pattern, insecure-avoidant infants revealed a positive value for the mean difference [old face-new face] FT, meaning that they tend to look less to the new face in comparison with the habituation face, and thus revealing less sensitivity to emotional change. Possibly using disengaging-deactivating strategies, infants classified as insecure-avoidant did not show the same sensitivity to emotional change, in contrast with infants classified as insecure-resistant (C), as they gave priority to familiar stimuli, which is by both theoretical and empirical work with adults (Niedenthal et al., 2002; Fraley et al., 2006).

Moreover, our results concerning infants’ visual preferences across the specific attachment sub-groups (secure, resistant, and avoidant), are similar to the tendency found in the adult literature (e.g., Mikulincer, 1998; Mikulincer et al., 2000, 2002; Niedenthal et al., 2002; Fraley et al., 2006; Dewitte et al., 2007; Dewitte and De Houwer, 2008; Edelstein and Gillath, 2008). According to adult *attachment theory*, individual differences in *attachment*-related anxiety reflect variations in individuals’ vigilance to cues relevant to appraising and monitoring the availability and responsiveness of significant others.

However, despite this study’s contributions, the sample size limits the generalization of our findings. Furthermore, since the assessment of both variables (infants’ attachment and the attention deployed to facial expressions of emotions) happened concurrently, we were not able to make inferences about the direction of the association found, which would be interesting to address prospectively. We must also note that in our design, pleasant and unpleasant expressions were, respectively, collinear with the emotional categories of happiness and anger, which precludes a conclusive interpretation of this factor. However, we hope that our findings come to be complemented by future work on the effects of affective properties across a broader range of emotional categories.

In conclusion, our results are consistent with the notion that variation in perceptual vigilance, and particularly the heightened vigilance to changes in emotional expression, may underlie attachment-related anxiety. It is noteworthy that our findings were generally consistent regardless of the *valence* and/or *arousal* (or emotional change resulting from the combination of both) of the emotions being perceived. In other words, highly resistant infants were just as sensitive to changes in positive emotions, such as happiness, as they were to negative emotions, such as anger, as well as to more or less intense emotions (e.g., High Angry Face vs. Low Angry Face). The heightened sensitivity to emotional change in insecure-resistant infants independent of emotional contrast (positive vs. negative, more vs. less intense) is an important finding because it suggests that in appraising the availability and responsiveness of the attachment figure the attachment system does not differentially weigh signals that are indicative of happiness (acceptance, sensitivity) vs. angriness (rejection, control) (see also Zhang and Hazan, 2002).

In this work, we have attempted to understand how different attachment patterns are linked to different types of emotional information processing. Future studies should proceed in advancing this line of research, expanding our findings across other age groups, and integrating different stimuli (e.g., both attachment and non-attachment related) and/or using different experimental protocols or paradigms (the importance of using multiple outcome measures in infant research was recently addressed by LoBue et al., 2020). Multimodal assessment of attention in infants using different tasks or paradigms may facilitate the identification of early behavioral and neurocognitive markers of risk factors for adverse developmental outcomes such as socioemotional difficulties.

## Data availability statement

The raw data supporting the conclusions of this article will be made available by the authors, upon request, without undue reservation.

## Ethics statement

The studies involving humans were approved by Comissão de Ética para a Saúde do Centro Hospitalar de São João e Comissão de Ética da Unidade Local de Saúde de Matosinhos. The studies were conducted in accordance with the local legislation and institutional requirements. Written informed consent for participation in this study was provided by the participants’ legal guardians/next of kin.

## Author contributions

JG: Conceptualization, Data curation, Formal analysis, Investigation, Methodology, Project administration, Resources, Software, Visualization, Writing – original draft, Writing – review & editing. MF: Funding acquisition, Investigation, Methodology, Project administration, Resources, Supervision, Writing – review & editing. SS: Data curation, Formal analysis, Investigation, Methodology, Software, Writing – review & editing. PL-d-S: Conceptualization, Formal analysis, Investigation, Supervision, Writing – review & editing. FF-S: Conceptualization, Data curation, Formal analysis, Methodology, Supervision, Writing – review & editing.

## Footnotes

^1^ http://www.smivision.com
