# Corrigendum: Differential effects of attachment security on visual fixation to facial expressions of emotion in 14-month-old infants: an eye-tracking study

**DOI:** 10.3389/fpsyg.2024.1430440

**Published:** 2024-05-23

**Authors:** Joana L. Gonçalves, Marina Fuertes, Susana Silva, Pedro Lopes-dos-Santos, Fernando Ferreira-Santos

**Affiliations:** ^1^Center for Research in Psychology for Positive Development, Lusíada University, Porto, Portugal; ^2^Center for Psychology at University of Porto, Faculty of Psychology and Education Sciences, University of Porto, Porto, Portugal; ^3^Escola Superior de Educação, Instituto Politécnico de Lisboa, Lisboa, Portugal; ^4^Neurocognition and Language Research Group, Center for Psychology at University of Porto, Faculty of Psychology and Education Sciences, University of Porto, Porto, Portugal; ^5^Faculty of Psychology and Education Science, University of Porto, Porto, Portugal; ^6^Laboratory of Neuropsychophysiology, Faculty of Psychology and Education Science, University of Porto, Porto, Portugal

**Keywords:** attachment, information processing, eye-tracking, affect-biased attention, facial expression of emotion, infancy

In the published article, there were errors in the affiliations listed for the first author Joana L. Gonçalves. Only affiliation 1 should be listed for this author, meaning that affiliations number 2, 3, and 4 should be eliminated from the first author's affiliation. Consequently, the affiliations previously listed as “3. inED Centre for Research and Innovation in Education, School of Higher Education, Polytechnic Institute of Porto, Porto, Portugal” and “4. Department of Social and Behavioral Sciences, University of Maia, Maia, Portugal” should be removed entirely from the affiliation list. The remaining affiliation numbers should be adjusted accordingly. The corrected author details are shown below.

“Joana L. Gonçalves^1*^, Marina Fuertes^2, 3^, Susana Silva^4^, Pedro Lopes-dos-Santos^2, 5^ and Fernando Ferreira-Santos^5, 6^

^1^ Center for Research in Psychology for Positive Development, Lusíada University, Porto, Portugal

^2^ Center for Psychology at University of Porto, Faculty of Psychology and Education Sciences, University of Porto, Porto, Portugal

^3^ Escola Superior de Educação, Instituto Politécnico de Lisboa, Lisboa, Portugal

^4^ Neurocognition and Language Research Group, Center for Psychology at University of Porto, Faculty of Psychology and Education Sciences, University of Porto, Porto, Portugal

^5^ Faculty of Psychology and Education Science, University of Porto, Porto, Portugal

^6^ Laboratory of Neuropsychophysiology, Faculty of Psychology and Education Science, University of Porto, Porto, Portugal

Additionally, in the published article, there was an error in the Funding statement. The original text “The author(s) declare financial support was received for the research, authorship, and/or publication of this article. This study was funded by national funds through FCT - Foundation for Science and Technology, I.P., under the Project “UIDB/04375/2020” and a doctoral grant awarded to the first author JG from the Portuguese Foundation for Science and Technology (SFRH/BD/90853/2012)” is not correct. The correct Funding statement appears below.

