# Supplementary material for: Differential effects of attachment security on visual fixation to facial expressions of emotion in 14-month-old infants: an eye-tracking study
Source: Front Psychol. 2024 Feb 1;15:1302657. doi: 10.3389/fpsyg.2024.1302657 (PMC10917067; doi:10.3389/fpsyg.2024.1302657)
Supplement: Supplementary file 1 [file Table_1.DOCX]

Supplemental Materials

Table A1

*Protocols for counterbalancing stimulus presentation (12 per actress, 24 per two actresses)*

| Participant | | 1 | 2 | 3 | 4 | 5 | 6 | 7 | 8 | 9 | 10 | 11 | 12 |
| --- | --- | --- | --- | --- | --- | --- | --- | --- | --- | --- | --- | --- | --- |
| Block1 | Trial 1 | A_r | A_l | A_r | A_l | V_r | V_l | V_r | V_l | AV_r | AV_l | AV_r | AV_l |
|  | Trial 2 | V_l | V_r | AV_l | AV_r | AV_l | AV_r | A_l | A_r | A_l | A_r | V_l | V_r |
|  | Trial 3 | AV_r | AV_l | V_r | V_l | A_r | A_l | AV_r | AV_l | V_r | V_l | A_r | A_l |
| Block 2 | Trial 1 | A_l | A_r | A_l | A_r | V_l | V_r | V_l | V_r | AV_l | AV_r | AV_l | AV_r |
|  | Trial 2 | V_r | V_l | AV_r | AV_l | AV_r | AV_l | A_r | A_l | A_r | A_l | V_r | V_l |
|  | Trial 3 | AV_l | AV_r | V_l | V_r | A_l | A_r | AV_l | AV_r | V_l | V_r | A_l | A_r |

A = Arousal, High Happy paired with Low Happy; V = Valence, Low Angry paired with Low Happy; AV = Arousal and Valence, High Angry paired with low Happy; r = Right; l = Left
